# Synthesis of Novel Cu(II), Co(II), Fe(II), and Ni(II) Hydrazone Metal Complexes as Potent Anticancer Agents: Spectroscopic, DFT, Molecular Docking, and MD Simulation Studies

**DOI:** 10.1021/acsomega.4c06202

**Published:** 2024-09-10

**Authors:** Eyüp Basaran, Hatice Gamze Sogukomerogullari, Muhammed Tılahun Muhammed, Senem Akkoc

**Affiliations:** † Department of Chemistry and Chemical Processing Technologies, Vocational School of Technical Sciences, Batman University, Batman 72060, Türkiye; ‡ Medical Services and Techniques Department, Vocational School of Health Services, Gaziantep University, Gaziantep 27310, Türkiye; § Faculty of Pharmacy, Department of Pharmaceutical Chemistry, Suleyman Demirel University, Isparta 32260, Türkiye; ∥ Faculty of Pharmacy, Department of Basic Pharmaceutical Sciences, Suleyman Demirel University, Isparta 32260, Türkiye; ⊥ Faculty of Engineering and Natural Sciences, Bahcesehir University, Istanbul 34353, Türkiye

## Abstract

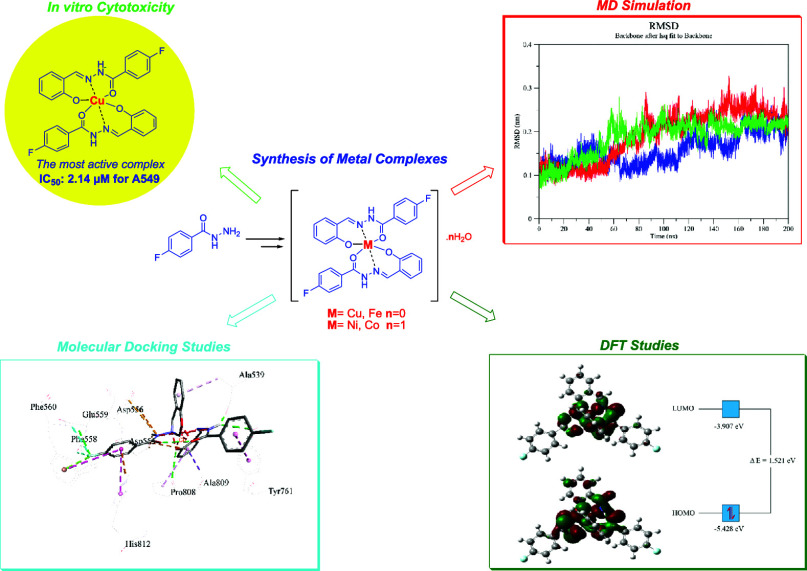

Metal complexes [FeL], [NiL]·H_2_O, [CuL], and [CoL]·H_2_O were formed by the ligand (**L**, 4-fluoro-*N*′-(2-hydroxybenzylidene)benzohydrazide) reacting with Fe(OAc)_2_, Ni(OAc)_2_·4H_2_O, Cu(OAc)_2_·H_2_O, and Co(OAc)_2_·4H_2_O. The produced compounds were characterized using a variety of methods, such as NMR, UV–vis, FT-IR, magnetic susceptibility, elemental analysis, and molar conductivity. The spectrum of the data indicates that the geometry of the complex molecular structures is octahedral with six coordination sites. The ligand and its different metal complexes were tested in a human lung cancer cell line and a normal embryonic kidney cell line. A cytotoxic assay revealed that **L-Cu** is the most potent chelate against cancer cell lines. A computational study was performed to rationalize this finding. The binding potential of relatively active compounds to a suitable target was analyzed. For this purpose, a target that is known to be inhibited by small compounds with a scaffold similar to that of the synthesized compounds, lysine-specific demethylase 1 (LSD1), was first determined. Molecular docking studies demonstrated that **L-Cu** has a high binding potential to LSD1 at a level comparable to that of a standard ligand. Molecular dynamics (MD) simulations revealed that **L-Cu** and **L** form stable complexes with the enzyme. Furthermore, the MD simulation study showed that **L-Cu** remained inside the binding pocket of the enzyme during the 200 ns simulation period. Density functional theory (DFT) studies demonstrated that the chemical stability of **L** was higher than that of its chelate form, **L-Cu**.

## 1 Introduction

Lung cancer is the primary cause of cancer-related deaths worldwide, with a mortality rate of approximately 18% in 2020.^1,2^ Lung cancer continues to escalate globally, imposing significant physical, emotional, and financial burdens on individuals, families, and societies.^2^ Platinum-derived chemotherapeutic agents, namely cisplatin, oxaliplatin, and carboplatin, have been widely utilized in the medical management of various malignancies, notably lung cancer.^3−5^ Nevertheless, platinum-based drugs exhibit significant toxicity and adverse events such as neurotoxicity, nephrotoxicity, and gastrointestinal responses, thereby constraining the feasible therapeutic dosage.^6−8^ Moreover, they can generate intrinsic or acquired resistance to drugs, posing a challenge to the effective management of cancer in clinical settings.^9,10^ Therefore, there is an urgent need for innovative cancer therapies.

The investigation of biologically important inorganic compounds is a growing field of research. Metals are essential for numerous biological processes and play pivotal roles.^11^ Cu, a trace metal, plays a significant role in numerous biochemical processes.^12,13^ For instance, copper serves as a catalytic cofactor that participates in redox and metabolic processes in living organisms.^14^ Cu(II) complexes play a crucial role in numerous enzymes owing to their diverse coordination geometries and catalytic characteristics.^15^ Currently, Cu(II) complexes are widely utilized as promising pharmacological agents for cancer treatment.^16^ Studies have indicated that cancer cells exhibit a higher preference for copper ions than for other metals, leading to the increased absorption of copper ions.^17,18^ However, Cu(II) complexes have been demonstrated to have a range of biological effects, including anticancer, antimicrobial, anti-inflammatory, and antioxidant characteristics.^19−24^

**Figure 1:**
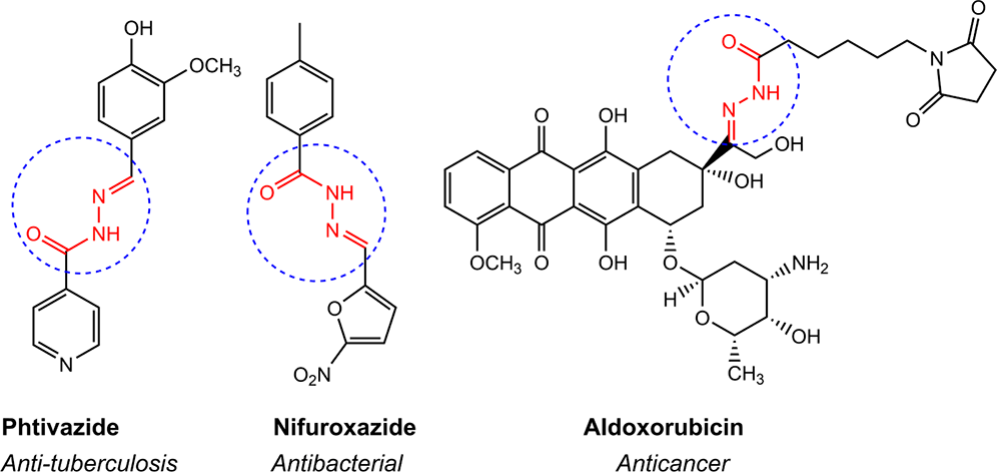
Marketed drugs containing *N*-acyl hydrazone derivatives with biological applications.

Hydrazones, a class of significant Schiff base ligands, exhibit diverse pharmacological properties, including anticancer, antioxidant, antimicrobial, antituberculosis, anti-inflammatory, antibacterial, and enzyme inhibitory effects.^25−29^ The ability of hydrazones to undergo enol–ketone interconversion enables them to form coordination complexes with metal-ion centers, either as anionic ligands or ketones.^30^ Various commercially available drugs incorporate *N*-acyl hydrazone derivatives,^31^ as depicted in Figure 1. Multiple studies have shown that medications containing the *N*-acyl hydrazone fragment are more effective than the original medicament. For instance, Effenberger et al.^32^ found that a doxorubicin hybrid, which had an *N*-acyl hydrazone moiety, exhibited more effective anticancer properties compared to unmodified doxorubicin, demonstrating a unique mechanism of action.

**Scheme 1:**
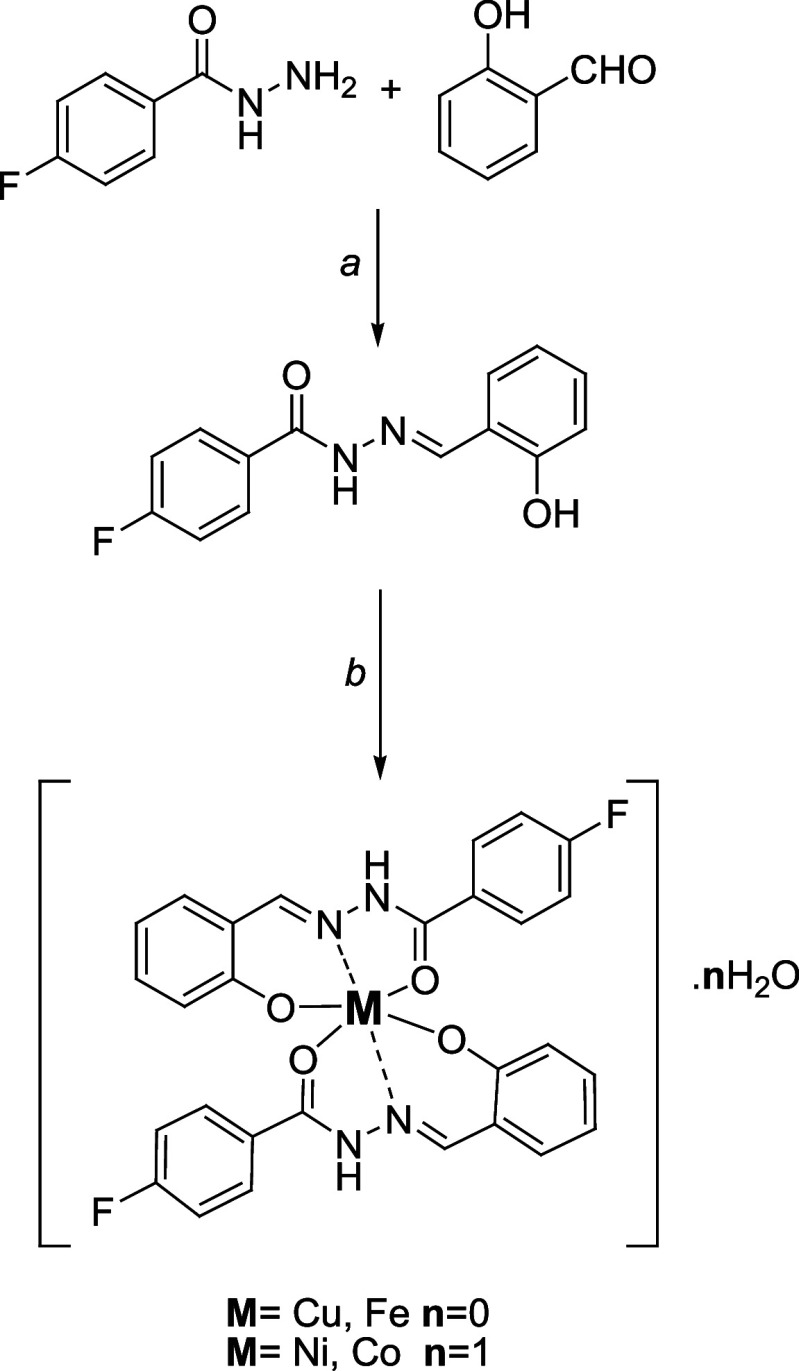
Synthesis of Novel Metal Complexes Reagents and Conditions: a) EtOH, Reflux, 4 h; b) EtOH/MeOH (1:1), 65 °C, 0.5 h

Based on the above advantages of hydrazones and their metal complexes, a hydrazide ligand and its associated Cu(II), Ni(II), Fe(II), and Co(II) complexes were used in this study (Scheme 1). A human lung cancer cell line and a normal embryonic kidney cell line were used to examine the compounds. **L-Cu** was shown to be the most effective chelate against cancer cell lines using the cytotoxic test. The structural and functional properties of the results were better understood, and their interpretation was improved by molecular docking, MD simulations, and DFT studies.

## 2 Results and Discussion

### 2.1 Characterization

The ligand (**L**) was synthesized by the condensation of salicylaldehyde and 4-fluorobenzoic hydrazide according to a previously reported method given in the literature.^33^**L** was used to produce metal complexes with copper(II), cobalt(II), nickel(II), and iron(II) acetate metal salts. Various analytical techniques such as elemental analysis, FT-IR, UV–vis spectroscopy, molar conductivity, and magnetic susceptibility measurements were used to characterize metal complexes (Figures S1–S12). All compounds were examined in the crystallization experiments; however, no single crystals were formed. Therefore, single-crystal X-ray examination could not be performed. The complexes formed by **L** with metal salts had a stoichiometric ratio of 1:2 (metal:ligand). A molar conductivity of 10^–3^ M at room temperature for the complexes was measured in DMF solvent. The complexes did not exhibit much electrolytic conductivity, as shown by molar conductivity studies ranging from 1.52 to 2.85 μS/cm.^34,35^

The FT-IR spectrum of **L** displays bands at 3193 [υ(O–H)], 3174 [υ(N–H)], 1650 [υ(C=O)], and 1605 [υ(C=N)] cm^–1^.^33^ Upon examination of the FT-IR spectra of the complexes, the O–H stretching band was observed as a broad and wide band within the range of 3202–3197 cm^–1^.^36^ The FT-IR spectra of all metal complexes showed an N–H stretching band within the range of 3105–3155 cm^–1^.^37^ This indicated the absence of keto–enol tautomerism in the structure. The C=O stretching band, which was found at 1650 cm^–1^ in **L**, was observed in the range of 1627–1600 cm^–1^ in the metal complexes. This indicated that the metal interacted with the carbonyl (C=O) group in the ligand to form complexes.^38^ Moreover, the C=N band associated with the azomethine group, first detected at 1605 cm^–1^ in **L**, was consistently found within the range of 1600–1562 cm^–1^ in all complexes. This suggests that the C=N group is the site of complexation in **L**-complexes.^39,40^ The newly observed bands within the wavelength range of 567–528 cm^–1^ were attributed to the M–N stretching band, whereas the newly observed bands within the range of 482–462 cm^–1^ were related to the M–O stretching band.^41−43^ At room temperature, substances were measured using UV–vis spectra in the 190–800 nm region in DMF solvent with a concentration of 2 × 10^–5^ M. The ligands exhibited n−π* transitions within the wavelength range of 338–330 nm and π–π* transitions within the range of 320–304 nm. The range of observed n−π* transitions in complexes is 392–342 nm, whereas the range of recorded π–π* transitions is 338–306 nm.^44,45^ Furthermore, the complexes exhibited additional transitions within the 400–498 nm range, which were not observed for the ligand. These changes are indicative of charge transfer.^46^

The magnetic susceptibility of the copper complex was 1.86 μ_B_. An octahedral geometry has been suggested for the **L-Cu** complex.^47^ The cobalt complex exhibited a magnetic susceptibility value of 4.81 μ_B_. This suggests that the **L-Co** complex had an octahedral structure. Room-temperature measurements of the cobalt(II) complex indicate magnetic moments of 4.81 μ_B_, indicating the high-spin character of the complexes.^48^ The magnetic susceptibility of the iron complex was 4.19 μ_B_. An octahedral structure was preferred for the **L-Fe** complex.

### 2.2 Cytotoxic Activity Studies

The complexes were tested in human lung cancer and normal embryonic kidney cell lines for 72 h. The results are presented in Table 1.

**Table 1:** IC_50_ Results for Compounds in Human Cell Lines

	IC_50_ (μM)	
compound	A549	HEK-293T
**L**	36.70	18.15
**L-Cu**	2.14	2.32
**L-Co**	59.23	17.77
**L-Fe**	78.79	69.17
**L-Ni**	N.T.^a^	N.T.^a^
cisplatin	4.23	2.62

a N.T.: not tested.

The copper metal complex was found to have a higher cytotoxic effect in the lung cancer cell line (A549) for 72 h in vitro compared to cobalt and iron metal complexes, with an IC_50_ value of 2.14 μM. The higher cytotoxic effect of the copper metal-based complex (**L-Cu**) in A549 cells compared to cobalt and iron metal complexes (**L-Co** and **L-Fe**) may be due to the stronger and more diverse effects of **L-Cu** on cellular mechanisms. This situation may be explained by factors such as the intracellular accumulation of **L-Cu**, oxidative stress production, its effect on genetic and epigenetic changes, and stronger activation of apoptosis and necrosis pathways. The cytotoxic activity of **L-Cu** in A549 cells might be important for the evaluation and development of new metal-based therapeutic agents for cancer treatment.

When the ligand and **L-Cu** were compared, the cytotoxic effect of **L-Cu** was almost 17 times more cytotoxic than that of the ligand, including 2-hydroxybenzylidene and 4-fluorobenzohydrazide groups. **L-Cu**, having a 4-fluorobenzohydrazide group, was approximately twice as effective as cisplatin in A549 cells. However, the effect of the other two metal complexes (**L-Co** and **L-Fe**) and the ligand used as the starting material on the growth of A549 cells was very low compared to that of the positive control drug. Moreover, as shown in Table 1, the effectiveness of **L** in inhibiting the growth of lung cancer cells was higher than that of both metal complexes (**L-Co** and **L-Fe**) prepared using this ligand. Cytotoxic activity studies of complex **L-Ni** could not be performed because this complex was not fully dissolved in 0.5% DMSO and was precipitated in DMEM.

**Figure 2:**
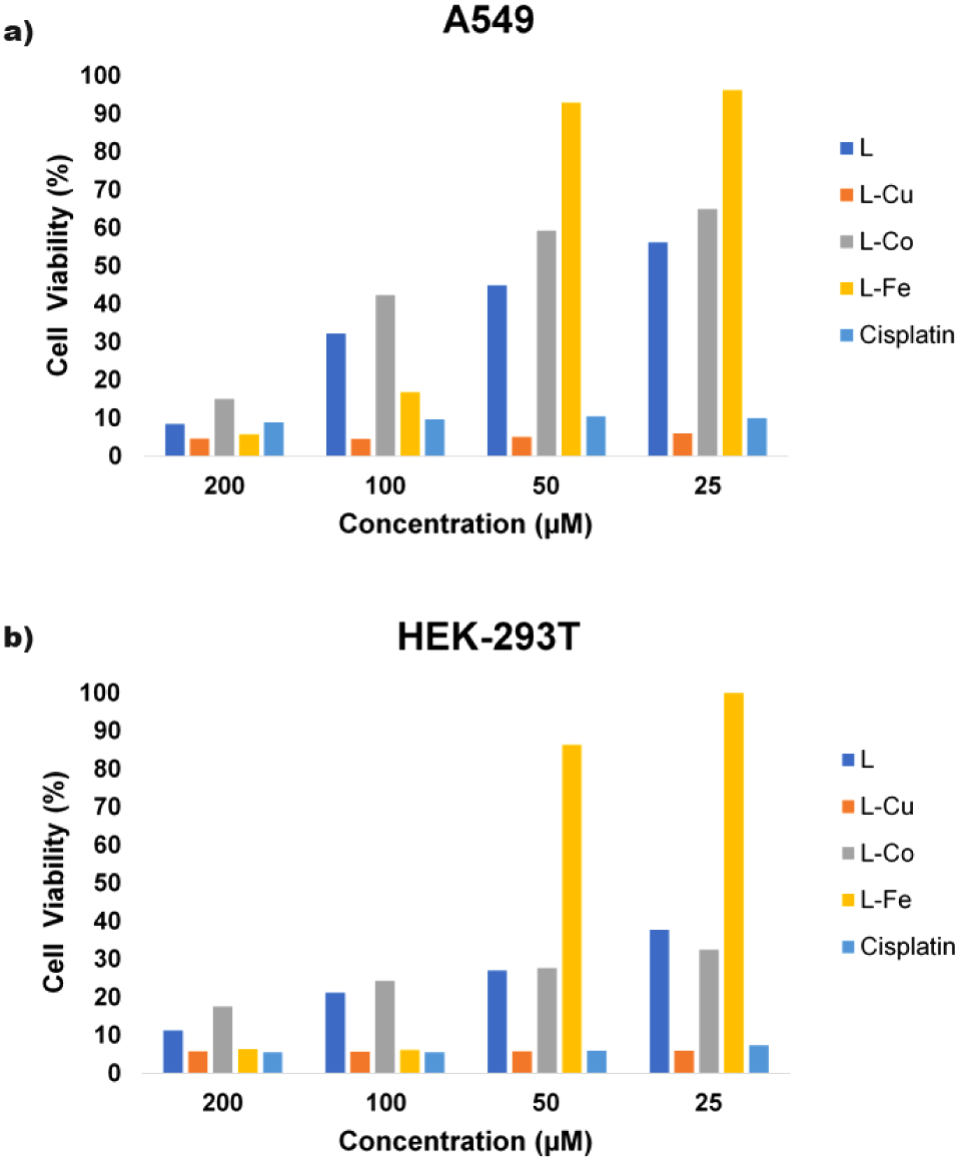
Cell viability ratio changes in the (a) A549 and (b) HEK-293T cell lines depending on the concentrations of the tested compounds.

The toxic effects of the two metal complexes (**L-Co** and **L-Fe**) and **L** on the healthy human embryonic kidney cell line were higher than their cytotoxic effects on the human lung cancer cell line. In particular, the selectivity of complex **L-Co** on healthy cells appeared to be low compared to that of the others. For example, when the complex **L-Co** was tested against the A549 cell line, the IC_50_ value was found to be 59.23 μM, whereas when it was tested against the HEK-293T cell line under the same experimental conditions, the IC_50_ value was 17.77 μM. It was found that the toxic effect values of **L** and complex **L-Co** on HEK-293T were close to each other, with IC_50_ values of 18.15 and 17.77 μM, respectively. Likewise, complex **L-Cu** and cisplatin, gave similar results on HEK-293T with IC_50_ values of 2.32 and 2.62 μM, respectively. The effect of the different metal complexes and ligands on the cells, depending on the concentrations studied, is shown in Figure 2.

Cell viability rates in A549 were very low at a 200 μM concentration of the compounds, such as 8.51%, 4.66%, 15.05%, 5.72%, and 8.98% for **L**, **L-Cu**, **L-Co**, **L-Fe**, and cisplatin, respectively. When the concentration of the compounds was reduced to 50 μM, an increase in A549 cell viability was observed and was determined as 44.90%, 5.10%, 59.32%, 92.96%, and 10.55% for **L**, **L-Cu**, **L-Co**, **L-Fe**, and cisplatin, respectively.

### 2.3 Rationale for Selecting LSD1 as the Target

**Figure 3:**
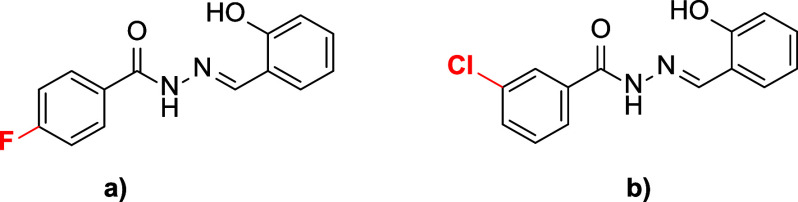
Structures of (a) **L** and (b) CHBH.

The in vitro cytotoxicity assay demonstrated that the copper chelate of **L** was the most active metal chelate. In addition, **L** exhibited moderate activity in both cancer cell lines. The probable mechanism of the anticancer activity was explored using molecular modeling. An appropriate target was selected for this purpose. A previous study reported that a compound with a structure similar to **L**, (3-chloro-*N*′-(2-hydroxybenzylidene)benzohydrazide (CHBH)), was found to halt cell proliferation in several human cancer cell lines. CHBH is a selective LSD1 inhibitor.^49^ As the two compounds have high structural similarity, **L** was also expected to be an LSD1 inhibitor (Figure 3). LSD1 is an essential demethylase that is involved in myeloid cell differentiation.^50^ The potential of **L** and **L-Cu** to bind to LSD1 and form a stable complex with the enzyme was investigated using molecular docking and MD simulations.

### 2.4 Molecular Docking

**Figure 4:**
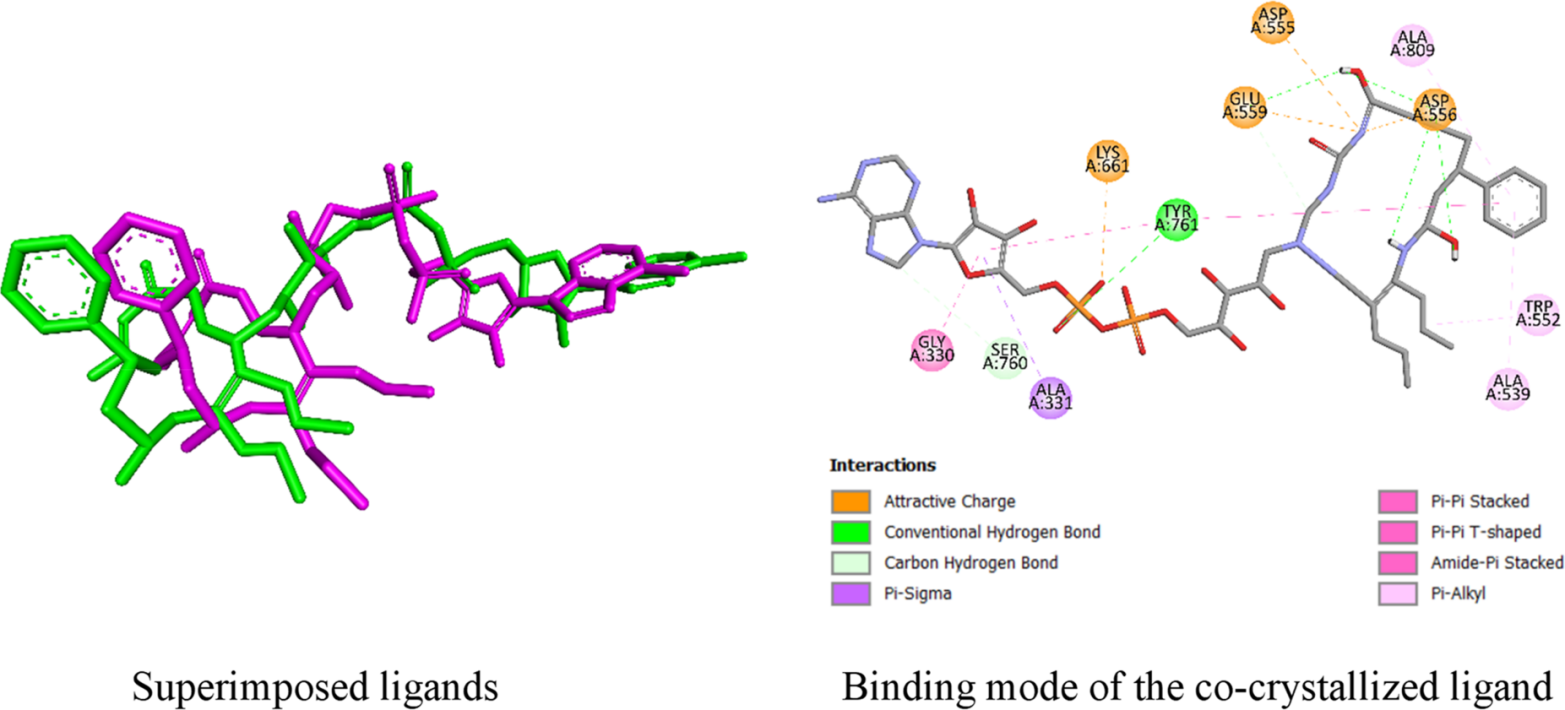
Superimposed crystal ligand (in green) and redocked ligand (in magenta) with their interaction profile to human LSD1 (6NQU).

The docking procedure was validated by redocking the two compounds with relatively high cytotoxic activity. To this end, the redocked cocrystallized ligand and its crystalline form were superimposed. The RMSD value was calculated to measure the potential of the redocked ligand to settle in the vicinity of the target enzyme. In turn, this confirmed the correctness of the grid box determined for the docking. The RMSD value was found to be 2.3931 Å (Figure 4). This value was higher than the ideal upper RMSD limit (2 Å).^51^ Additionally, the binding potential of the cocrystallized ligand to the LSD1 enzyme was considered in pursuing a docking study using this procedure. The ligand strongly interacted with a relatively good binding affinity. It formed five conventional hydrogen bonds and 12 other types of interactions with the enzyme (Table 2 and Figure 4). The binding energy was −9.7 kcal/mol (Table 2). The ligand was found to have a good binding potential for the enzyme. The interaction potential of the cocrystallized ligand is promising for reliable docking studies. Furthermore, the stability of the enzyme–compound complexes was measured using MD simulations. Hence, the docking study was performed using the same procedure.

**Table 2:** Binding Energy and Binding Residues of Compounds **L**, **L-Cu**, and the Co-crystallized Ligand in their interaction with LSD1

Compounds	Binding energy (kcal/mol)	Conventional hydrogen bond residues	Other interaction residues
ligand	–9.7	Asp556(3), Glu559, Tyr761	Gly330^a^, Ala331^b^, Ala539^c^, Trp552^c^, Asp555^d^, Asp556^d^, Glu559^d^, Lys661^d^, Ser760^e^, Tyr761(2)^a^, Ala809^c^
**L**	–8.7	Ser760, Val811	Arg316^c^, Arg316^f^, Ala331^c^, Leu659^c^, Trp751^a^, Thr810^e^, Val811^c^
**L-Cu**	–13.3	Ala539, Glu559, Tyr761, Pro808	Ala539^c^, Asp555^d^, Asp556(2)^d^, Phe558^f^, Phe560^a^, Phe560^e^, Phe560^f^, Tyr761^a^, Pro808^c^, Ala809^b^, His812^a^, His812^g^

a pi–pi.

b pi–sigma.

c pi–alkyl.

d Attractive charge.

e Carbon–hydrogen.

f Halogen.

g pi–sulfur.

The potential of **L** and its most active chelate, **L-Cu**, to bind to LSD1 was explored through molecular docking. The binding potential of **L-Cu** was higher than that of **L**. The intermolecular interaction binding energy of **L-Cu** (−13.3 kcal/mol) was much lower than that of **L** (−8.7 kcal/mol). Therefore, **L-Cu** was expected to have a higher binding affinity for LSD1. **L-Cu** forms four conventional hydrogen bonds with the enzyme, whereas **L** forms only two conventional hydrogen bonds with the enzyme (Table 2 and Figure 4). Similarly, compound **L-Cu** had many more other types of interactions (13) than compound **L** (7). Hence, **L-Cu** was expected to have a much stronger interaction with the enzyme. The lower binding energy and stronger interaction of **L-Cu** imply a better binding potential. Therefore, it exhibited a higher binding potential for LSD1 (6NQU). Cytotoxic activity assays showed that **L-Cu** was more potent than **L**. The computational study results confirmed this finding, as **L-Cu** was found to have a greater inhibitory activity on LSD1.

**Figure 5:**
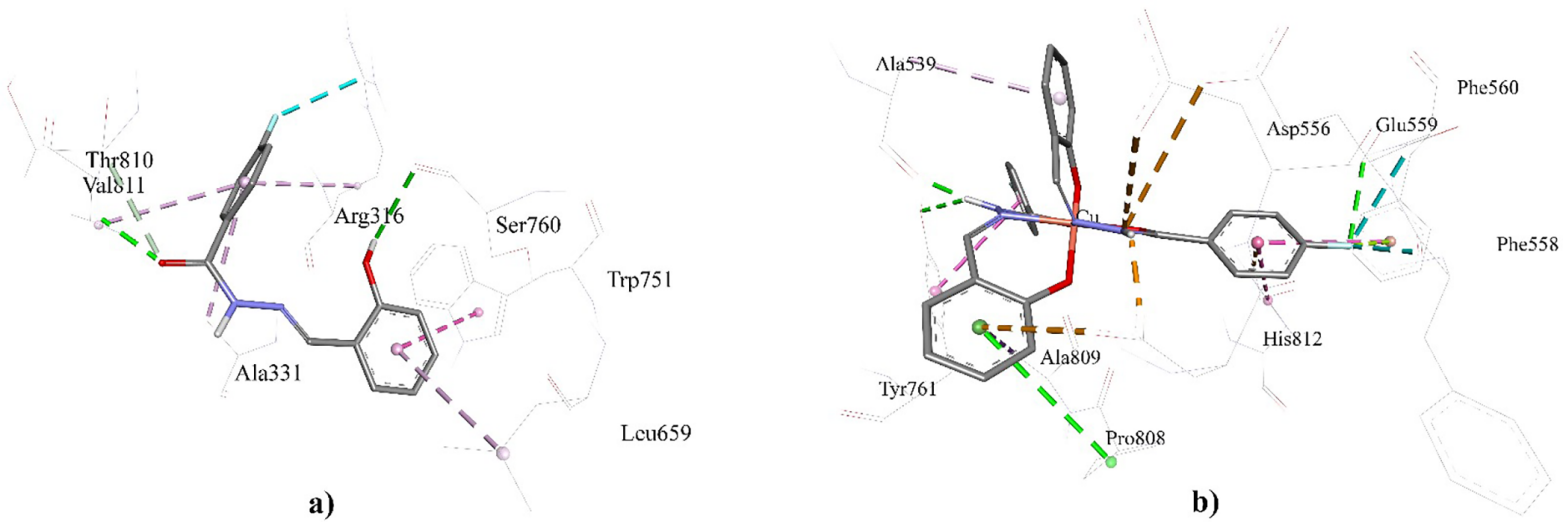
Binding profiles of (a) **L** and (b) **L-Cu** to LSD1.

The interaction of **L** with LSD1 was achieved via two conventional hydrogen bonds and seven other interaction types. In the conventional hydrogen bond (HB) formed with Ser760, **L** is the H-donor, whereas the amino acid is the H-acceptor. On the other hand, in the HB with Val811, the amino acid was the H-donor, whereas **L** was the H-acceptor. Similarly, in the carbon hydrogen bond formed with Thr810, the amino acid was the H-donor. The hydrophobic interactions are formed by the interaction of the pi-orbitals of **L** and the alkyl groups of the corresponding amino acid residues. The pi–pi interactions were formed with the involvement of the pi orbitals of both the components. The hydroxyl group substituent and carbonyl of the hydrazide group in its structure played a crucial role in HB formation (Figure 5). The hydrophobic interactions were achieved through the participation of the pi-orbitals of the phenyl rings of **L** (Figure 5a). During the interaction of **L-Cu** with the target, three HBs were formed with Ala539, Tyr761, and Pro808. **L-Cu** was an H-donor in these interactions, and the corresponding amino acids were H-acceptors. The amino acid residue was the H-donor in the HB formed with Glu559. Structure-interaction mechanism analysis of **L-Cu** revealed that NH in the hydrazide group played a crucial role in HB formation, as two of them were achieved by its involvement (Figure 5b). Attractive charge interactions occurred with the involvement of the positive charge on **L-Cu**. The hydrophobic interaction pattern of **L-Cu** was similar to that of the **L** interactions (Figure 5).

The compound that showed the highest activity in the wet-lab study and the highest binding potential toward LSD1 might have a higher binding potential relative to the cocrystallized ligand. **L-Cu** has a lower binding energy than the cocrystallized ligand, implying a higher binding potential (Table 2). However, the binding strengths of these two compounds were similar. In this respect, the cocrystallized ligand formed five conventional hydrogen bonds and 12 other types of interactions with the enzyme. Similarly, **L-Cu** formed four conventional hydrogen bonds and 13 other types of interactions. Since more hydrogen bonding was observed in the interaction of the cocrystallized ligand, it might have a slightly stronger interaction than that of **L-Cu**. In short, **L-Cu** is expected to have a better binding affinity toward the enzyme, whereas the cocrystallized ligand is expected to interact more strongly with a slim difference. In addition, **L-Cu** and its ligand interacted with similar residues, to some extent. In this regard, two conventional hydrogen bonds were formed via the same residues (Glu559 and Tyr761). Similarly, almost half of the other types of interactions occur via common amino acid residues (Ala539, Asp555, Asp556, Tyr761, and Ala809). In short, **L-Cu** exhibited a high binding potential for LSD1, which was comparable to that of the cocrystallized ligand.

### 2.5 MD Simulations

**Figure 6:**
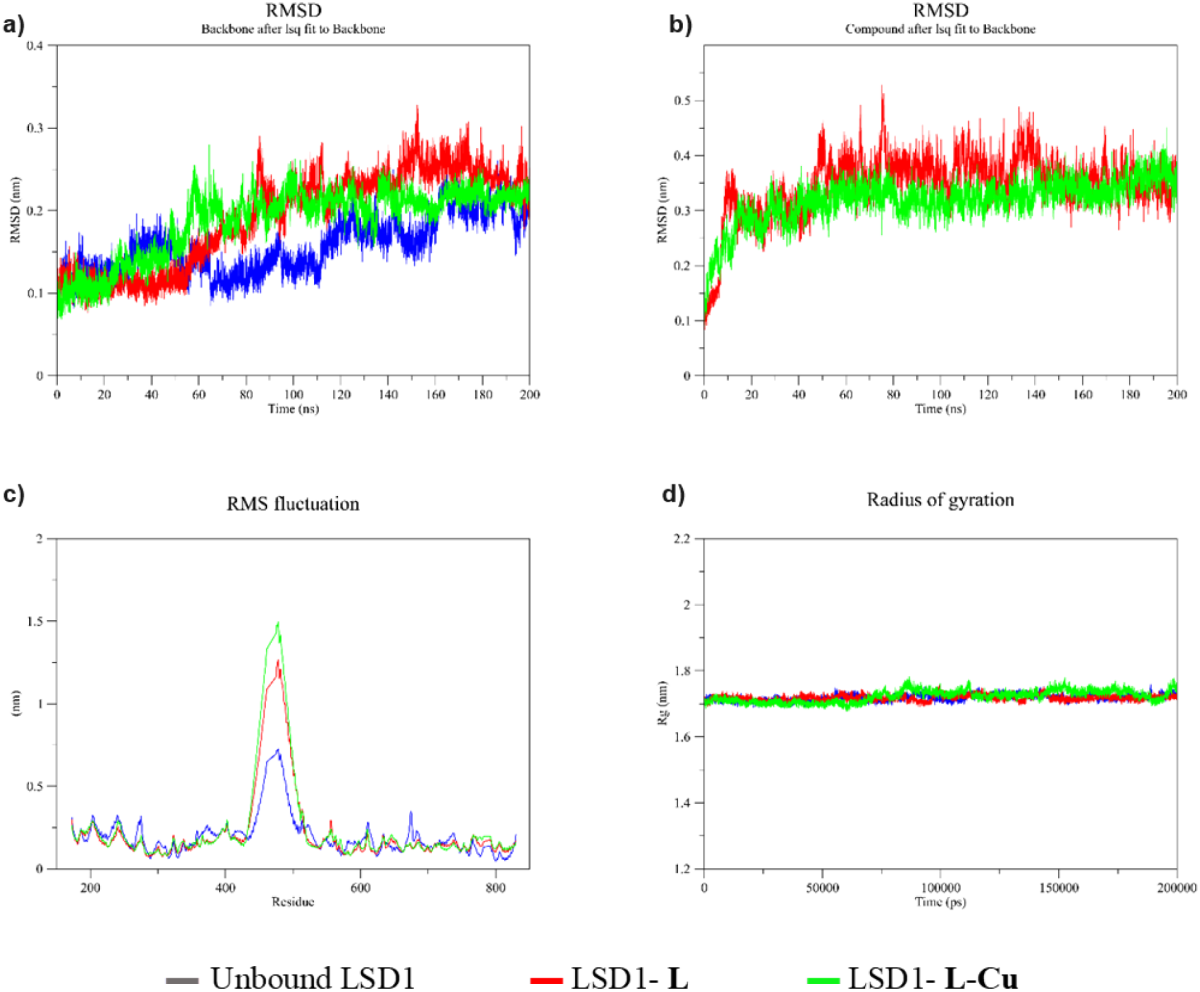
Protein (a) RMSD, (b) compound RMSD, (c) RMSF, and (d) *R*_g_ plots from the MD simulation.

MD simulations of the LSD1–compound complex were performed, and the resulting plots were compared to unbound enzyme plots. For this purpose, the backbone RMSD of the complexes and unbound enzyme, ligand RMSD of the complexes, RMSF, and *R*_g_ plots were sketched, and the resulting values were analyzed accordingly. RMSD plots of the backbone enzyme were drawn for the complexes and compared to the plot of the unbound enzyme to measure the changes in enzyme stability by ligand binding.^52^ The stability of the **L-Cu**-containing complex was similar to that of the unbound enzyme during the 200 ns simulation period. During the first 50 ns, all three structures exhibited similar RMSD plots. The unbound enzyme gave a relatively lower RMSD value in the 60–160 ns time interval. The **L-Cu**-containing complex and the unbound enzyme had similar RMSD values after 160 ns. In general, the structures gave an RMSD value below 0.3 nm (3 Å) during the 200 ns simulation period. Changes in RMSD values were observed; however, the changes remained within an acceptable range (Figure 6a). Hence, the stability of the enzyme does not disrupt the binding of the compounds.

Ligand RMSD plots were drawn to comprehend the status of the compounds relative to the binding site during the simulation period. **L-Cu** remained inside the enzyme binding site during the simulation period. Both compounds were stable for the first 12 ns. Thereafter, they had a relatively higher stability until the end of the 200 ns simulation. In addition, compound **L** exhibited higher changes in its RMSD value, implying greater movement inside the binding site during the simulation period (Figure 6b). Therefore, **L-Cu** was expected to have a higher probability of remaining inside the binding site. The higher number of hydrogen bonds formed by **L-Cu** may have played a crucial role in maintaining the compound inside the binding site. Hydrogen bonding plays a well-known role in the binding of compounds to their targets and their maintenance inside the binding site.^53^

The RMSF plot was used to measure per-residue perturbations in the enzyme structure. The *R*_g_ plot has also been used to measure the compactness of an enzyme or enzyme–compound complex.^54^ The RMSF plots of the three structures were similar. A significant change was observed in the 432–520 amino acid interval, with the enzyme–**L-Cu** complex having the highest variation (Figure 6c). The *R*_g_ values of the structures were similar to nearly 1.72 nm on average. The **L**-containing complex and unbound enzyme exhibited almost the same *R*_g_ plot during the simulation period. On the other hand, the **L-Cu**-containing complex gave the lowest *R*_g_ value at some time intervals, such as in the 12–70 ns interval, and also the highest *R*_g_ value in some other time intervals, such as in the 82–112 and 145–181 ns intervals. In addition, the changes in *R*_g_ values were not high (Figure 6d). Hence, a significant difference in the stiffness of the three structures was not expected. To wrap up, the MD simulation demonstrated that the LSD1–compound complex would be stable. In addition, the LSD1–**L-Cu** complex was expected to have higher stability and the ligand had a higher probability of remaining inside the binding site.

### 2.6 DFT Studies

DFT analysis revealed various quantum-related electrical properties of the compounds. The status of **L** and its chelate form, **L-Cu**, was then compared to understand the changes caused by chelating the compound. For this purpose, MEP maps and FMOs were drawn and analyzed.

### 2.7 MEP Appraisal

**Figure 7:**
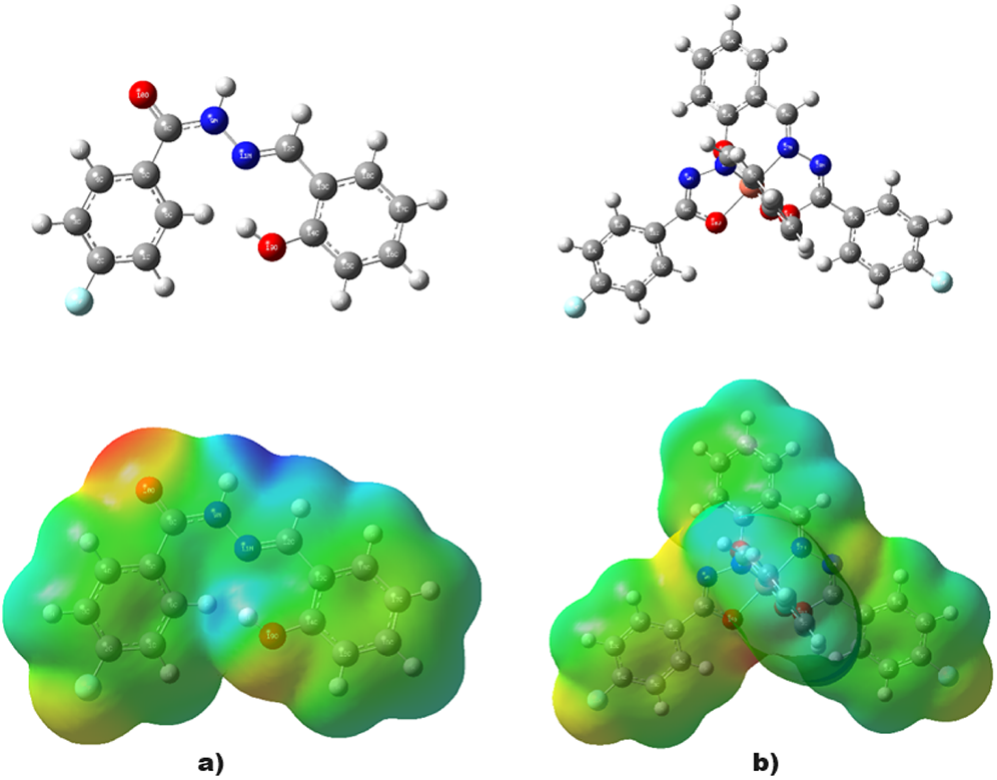
Optimized structures (the above panel) and the MEP maps (the below panel) of (a) **L** and (b) **L-Cu**.

The MEP of a compound is generated by its electrons and nuclei, which define its electron-rich and electron-poor parts. Hence, it facilitates the reactivity analysis by providing a charge distribution. In the MEP distribution maps, red and yellow regions depict electrophilic reactivity, whereas blue regions depict nucleophilic reactivity.^55^ The MEP map of **L** shows predominantly red regions near the oxygen of the hydroxyl functional group substituted with the benzylidene ring and the oxygen of the carbonyl group on the benzohydrazide ring. In addition, a predominantly yellow region was observed near the fluorine substituent on the benzohydrazide ring (Figure 7a). Similarly, predominantly yellow and red regions were observed near the oxygen at the junction of the molecule and copper, as well as near the fluorine substituent of the benzohydrazide ring (Figure 7b). Hence, the aforementioned vicinities may be the source of the electrophilic reactivity of the two compounds. The predominant blue regions were observed near the hydrogen atoms of the two compounds. Similarly, a predominantly blue region was observed around some nitrogen atoms in the two compounds, which is explicit for **L**. These vicinities were expected to result in the nucleophilic reactivity of the compounds.

### 2.8 FMO Analysis

**Figure 8:**
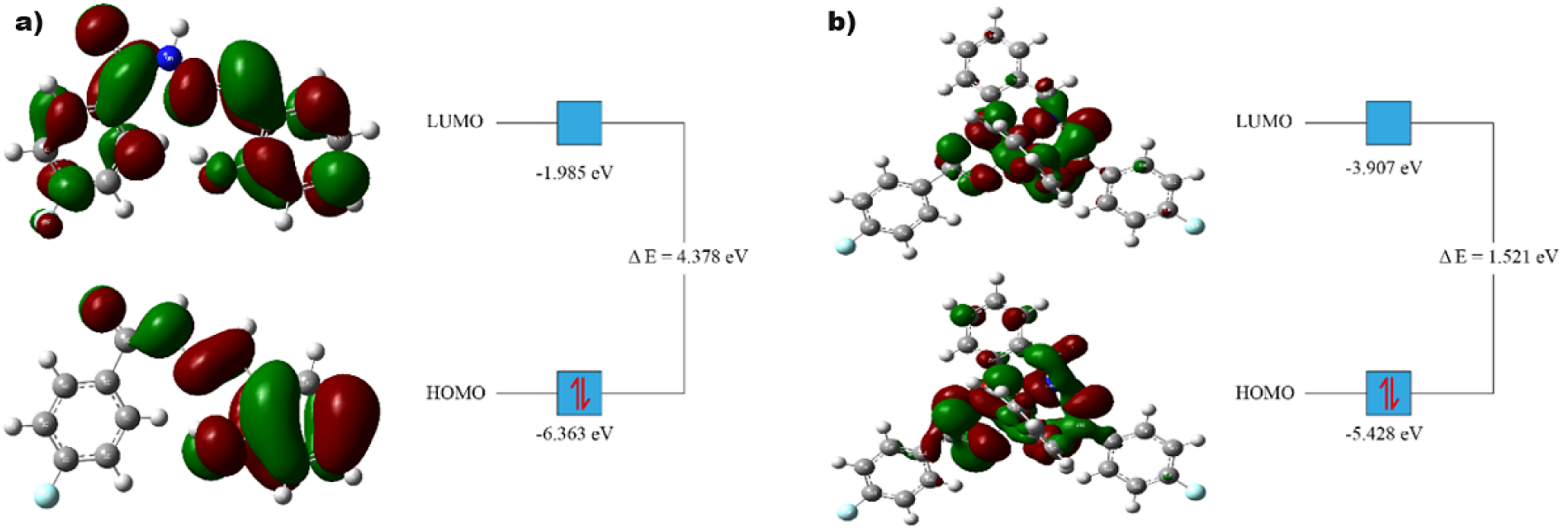
HOMO and LUMO distributions of (a) **L** and (b) **L-Cu** with their energy values as well as energy gaps at DFT/B3LYP/6-311G(d,p).

The relative stabilities of **L** and **L-Cu** were analyzed by energy calculations. The values are given in eV units, except for maximum charge transfer. The energy gap (Δ*E*) between the LUMO and HOMO energies of compounds is widely used to compare their relative chemical stability. A higher energy gap correlates with higher stability.^56^ The DFT study showed that the energy gap of **L** was much higher than that of its chelate form, implying higher chemical stability (Table 3 and Figure 8). The global hardness is also correlated with the atom’s resistance to electron transfer. **L** had a much higher hardness value, which most likely implies less reactivity, confirming its higher stability (Table 3). Therefore, **L** is anticipated to have a higher chemical stability and lower reactivity than **L-Cu**. This was also observed in the docking study. In the docking study, intramolecular interactions between the copper atom and the molecule were observed. Some of the intramolecular interactions were unfavorable, unlike the intermolecular interactions in **L-Cu** (Figure 5). This may affect the chemical stability of the chelate.

**Table 3:** HOMO, LUMO, and Related Energies of **L** and **L-Cu** at DFT/B3LYP/6-311G(d,p) (in eV)

Parameters	**L**	**L-Cu**
*E*_total_	–24 482.0	–93 524.5
*E*_HOMO_	–6.363	–5.428
*E*_LUMO_	–1.985	–3.907
Δ*E*	4.378	1.521
ionization potential (IP = −*E*_HOMO_)	6.363	5.428
electron affinity (*A* = −*E*_LUMO_)	1.985	3.907
chemical potential (μ = −(*I* + *A*)/2)	–4.174	–4.668
hardness (η = (*I* – *A*)/2)	2.189	0.761
Mulliken electronegativity (χ = (*I* + *A*)/2)^57^	4.174	4.668
softness (*S* = 1/2η)	0.228	0.657
electrophilicity index (ω = μ^2^/2η)^58^	3.972	14.316
maximum charge transfer (Δ*N*_max_ = (*I* + *A*)/2(*I* – *A*))^59^	0.953	3.067

The HOMO and LUMO distributions of the two compounds showed similarities and to some extent, differences between the two compounds. Some differences were also observed between the HOMO and LUMO levels of the two compounds. The HOMOs of compound **L** were mainly concentrated around the benzylidene and hydrazide bridges. On the other hand, LUMOs were observed around the benzylidene ring, between the hydrazide bridge, and around the 4-fluorophenyl ring (Figure 8a). The HOMOs of **L-Cu** are mainly concentrated around the entire structure except for the 4-fluorophenyl ring. On the other hand, a lower concentration of LUMOs was observed around not only the 4-fluorophenyl ring but also the phenyl group of the 2-hydroxybenzylidene ring of **L-Cu** (Figure 8b).

## 3 Conclusion

A hydrazone ligand and its associated Cu(II), Co(II), Fe(II), and Ni(II) (**L-Cu**, **L-Co**, **L-Fe**, and **L-Ni**) complexes were produced and characterized using a range of techniques, including elemental analysis, FT-IR, NMR, UV–vis, molar conductivity, and magnetic susceptibility. According to the data spectrum, the complex chemical structures had six coordination sites and octahedral shapes. Electrolytic conductivity was not observed for any of the compounds. The ligand and four different metal complexes were tested in the A549 and HEK-293T cell lines. The MTT assay results showed that complex-based copper had a very high cytotoxic effect on the A549 cell line after 72 h. A docking study revealed that the binding potential of **L-Cu** to LSD1 was comparable to that of the cocrystallized inhibitor. Compound **L** also had a binding potential but was lower than that of **L-Cu**. The resulting enzyme–compound complexes were found to be stable according to the MD simulation analysis. In addition, compound **L-Cu** was anticipated to remain inside the binding site during the 200 ns simulation period. A DFT study indicated that chelate formation decreased the chemical stability of **L**.

## 4 Experimental Section

### 4.1 Chemistry

#### 4.1.1 Synthesis of Ligand (**L**)

4-Fluoro-*N*′-(2-hydroxybenzylidene)benzohydrazide (**L**) was synthesized by reacting 4-fluorobenzoic hydrazide with salicylaldehyde under suitable conditions according to a previously reported method.^33^ A solution of 1 mmol of salicylaldehyde in ethanol was added to a solution of 1 mmol of 4-fluorobenzoic hydrazide in 50 mL of ethanol. The mixture was refluxed on a water bath for 4 h. After cooling the mixture, the precipitate was filtered, dried, and recrystallized from ethanol (Scheme 1).

White solid, yield: 79%, mp: 191–192 °C. FT-IR (υ_max_): 3193 (O–H), 3174 (N–H), 3068, 3038 (C–H), 1650 (C=O), and 1605 (C=N) cm^–1^. ^1^H NMR (400 MHz, DMSO-*d*_6_): δ 12.13 (s, 1H, NH), 11.26 (s, 1H, OH), 8.64 (s, 1H, CH=N), 8.06–7.99 (m, 2H, ArH), 7.55 (d, *J* = 7.6 Hz, 1H, ArH), 7.40 (d, *J* = 8.7 Hz, 2H, ArH), 7.30 (t, *J* = 7.7 Hz, 1H, ArH), 6.96–6.92 (m, 2H, ArH) ppm. ^13^C NMR (100 MHz, DMSO-*d*_6_): δ 165.98, 163.50, 162.23, 157.92, 148.75, 131.89, 130.90, 130.80, 129.92, 129.77, 119.82, 119.13, 116.88, 116.14, 115.92 ppm. UV–vis (DMF) λ_max_ (abs): 304 (0.433), 320 (0.600), 330 (0.668), 338 (0.605) nm; elemental analysis for C_14_H_11_FN_2_O_2_: C, 65.11; H, 4.29; N, 10.85%. Found: C, 64.45; H, 4.37; N, 10.54%.

#### 4.1.2 Synthesis of Metal Complexes

The complexes were synthesized by combining a solution of M(OAc)_2_·*n*H_2_O (0.25 mmol) (M = Cu, Co, Ni, and Fe; *n* = 1, 4, 4, 0) in MeOH with a solution of the ligand (**L**) (0.129 g, 0.5 mmol) in EtOH, using a 1:2 mol ratio of metal to ligand. The reaction mixture was refluxed for a duration of 30 min at 65 °C. The final product was filtered, purified using cold methanol and Et_2_O, and subsequently dried under vacuum, as shown in Scheme 1.

##### 4.1.2.1 [CuL_2_]

Color: green. Yield: 0.100 g (69%). 300 °C dec IR, (ATR) υ, cm^–1^: 3105 (N–H), 3066, 3024 (C–H), 2987 (C–H)alp, 1627 (C=O), 1600 (C=N), 528 (M–N), 482 (M–O); UV–vis (DMF) λ_max_ (abs): 308 (0.291), 318 (0.356), 332 (0.358), 358 (0.162), 370 (0.267), 392 (0.425), 404 (0.396) nm; μ_eff_: 1.86 μ_B_ Λ_M_ (10^–3^ M, in DMF, μS/cm): 1.89. Anal. Calc. for C_28_H_18_CuF_2_N_4_O_4_ (576.01); C, 58.38; H, 3.15; N, 9.73. Found: C, 58.25; H, 3.32; N, 9.65%.

##### 4.1.2.2 [NiL_2_]·H_2_O

Color: green. Yield: 0.51 g (35%). 290 °C dec IR, (ATR) υ, cm^–1^: 3202 (O–H), 3143 (N–H), 3062, 3008 (C–H)_arom_, 2985 (C–H)_alp_, 1604 (C=O), 1562 (C=N), 567 (M–N), 478 (M–O); UV–vis (DMF) λ_max_ (abs): 308 (0.358), 320 (0.371), 334 (0.408), 344 (0.373), 368 (0.239), 418 (0.218) nm; μ_eff_: 2.85 μ_B_ Λ_M_ (10^–3^ M, in DMF, μS/cm): 1.52. Anal. Calc. for C_28_H_20_F_2_N_4_NiO_5_ (589.17); C, 57.08; H, 3.42; N, 9.51. Found: C, 57.30; H, 3.42; N, 8.96%.

##### 4.1.2.3 [CoL_2_]·H_2_O

Color: orange. Yield: 0.78 g (53%). 300 °C dec IR, (ATR) υ, cm^–1^: 3197 (O–H), 3122(N–H), 3055, 3012 (C–H)_arom_, 2995 (C–H)_alp_, 1604 (C=O), 1566 (C=N), 555 (M–N), 466 (M–O); UV–vis (DMF) λ_max_ (abs): 304 (0.505), 320 (0.619), 328 (0.627), 342 (0.548), 358 (0.338), 380 (0.372), 404 (0.457), 424 (0.417) nm; μ_eff_: 4.81 μ_B_ Λ_M_ (10^–3^ M, in DMF, μS/cm): 2.85. Anal. Calc. for C_28_H_20_CoF_2_N_4_O_5_ (589.41); C, 57.06; H, 3.42; N, 9.51. Found: C, 57.23; H, 3.40; N, 9.10%.

##### 4.1.2.4 [FeL_2_]

Color: black. Yield: 0.109 g (76%). 285 °C dec IR, (ATR) υ, cm^–1^: 3155 (N–H), 3062, 3012 (C–H)_arom_, 2974 (C–H)_alp_, 1600 (C=O), 1562 (C=N), 536 (M–N), 462 (M–O); UV–vis (DMF) λ_max_ (abs): 306 (0.921), 316 (1.056), 322 (0.935), 352 (0.801), 368 (0.838), 392 (0.591), 400 (0.400), 444 (0.222), 498 (0.118) nm; μ_eff_: 4.19 μ_B_ Λ_M_ (10^–3^ M, in DMF, μS/cm): 2.28. Anal. Calc. for C_28_H_18_F_2_FeN_4_O_4_ (568.31); C, 59.18; H, 3.19; N, 9.86. Found: C, 59.48; H, 3.38; N, 9.57%.

### 4.2 Cytotoxic Activity Studies

Cytotoxic activity studies of compounds (**L**, **L-Cu**, **L-Co**, and **L-Fe**) were conducted according to the literature.^60^ Human lung epithelial carcinoma cells (A549; ATCC CCL-185) and healthy human embryonic kidney cells (HEK-293T) were cultured in Dulbecco’s modified Eagle’s medium-high-glucose supplemented with 10% fetal bovine serum and 1% GlutaMAX in a CO_2_ incubator at 37 °C. Cells seeded in 96-well plates were exposed to 200, 100, 50, and 25 μM of compounds for 72 h. The MTT stock solution (50 μL, 5 mg/mL) was added to each well. Absorbance was measured using an Epoch 2 ELISA plate reader at 590 nm. The IC_50_ values were calculated using GraphPad Prism Software version 5.

### 4.3 Molecular Docking

The crystal structure of lysine-specific demethylase 1 (LSD1) was obtained from the RCSB Protein Data Bank (PDB). The enzyme structure (PDB code: 6NQU) with a resolution of 2.70 Å contains a cocrystallized ligand, GSK2879552.^61^ The compound structures were drawn using ChemDraw Ultra and optimized using the Gaussian program.^62,63^ Protein and compound structures were prepared as previously described in earlier studies.^64^ The grid box was specified based on the position of the cocrystallized ligand. Molecular docking was performed using AutoDock Vina.^65^ The docking process was validated by redocking the cocrystallized ligand with the target enzyme. The docking results were visualized using Biovia Discovery Studio.^66^

### 4.4 MD Simulation

The stability of the LSD1–compound complexes obtained from docking was assessed through MD simulation to compare it with the stability of the unbound enzyme. Compound structure topologies were generated using the CGenFF server.^67^ Enzyme topologies were generated using appropriate GROMACS commands, as explained in the literature.^68,69^ After the system requirements were ready, the MD simulation was run for a 200 ns period. From the MD simulations, root-mean-square deviation (RMSD), root-mean-square fluctuation (RMSF), and radius of gyration (*R*_g_) plots were drawn using QtGrace, and the necessary analysis was performed based on the resulting plots.

### 4.5 DFT Studies

The DFT study of **L** and its active chelate **L-Cu** was performed using a Gaussian program.^70^ For this purpose, the two compounds were optimized on a 6-311G basis set using the DFT/B3LYP method in the ground state. Then, energy computation was performed using the same setup. The total, highest occupied molecular orbital (HOMO), and lowest unoccupied molecular orbital (LUMO) energies were obtained as atomic units (a.u.). The retrieved figures were converted to eV (electron volts). Thereafter, the related parameters were computed in eVs based on the HOMO and LUMO energies. In addition, molecular electrostatic potential (MEP) maps and frontier molecular orbitals (FMOs) were drawn and analyzed afterward.^71^

## Supporting Information Available

Brands and models of devices used in the study; FT-IR, UV–vis, and ^1^H and ^13^C NMR spectra of compounds (PDF)
