# Supplementary material for: Synthesis of Novel Cu(II), Co(II), Fe(II), and Ni(II) Hydrazone Metal Complexes as Potent Anticancer Agents: Spectroscopic, DFT, Molecular Docking, and MD Simulation Studies
Source: ACS Omega. 2024 Sep 10;9(38):40172–81. doi: 10.1021/acsomega.4c06202 (PMC11425624; doi:10.1021/acsomega.4c06202)
Supplement: Supplementary file 1 — ao4c06202_si_001.pdf [file ao4c06202_si_001.pdf]

Supporting Information

Synthesis of novel Cu(II), Co(II), Fe(II), and Ni(II) hydrazone metal complexes as potent anticancer agents: Spectroscopic, DFT, molecular docking, and MD simulation studies

Eyüp Başaran[a], Hatice Gamze Sogukomerogullari[b*], Muhammed Tılahun Muhammed[c], Senem Akkoc[d,e*]

[a]*Department of Chemistry and Chemical Processing Technologies, Vocational School of Technical Sciences, Batman University, Batman, 72060, Türkiye.*
[b]*Medical Services and Techniques Department, Vocational School of Health Services, Gaziantep University, Gaziantep, 27310, Türkiye.*
[c]*Suleyman Demirel University, Faculty of Pharmacy, Department of Pharmaceutical Chemistry, Isparta, 32260, Türkiye.*
[d]*Suleyman Demirel University, Faculty of Pharmacy, Department of Basic Pharmaceutical Sciences, Isparta 32260, Türkiye.*
[e]*Bahcesehir University, Faculty of Engineering and Natural Sciences, Istanbul, 34353, Türkiye.*

***Corresponding Authors:** Tel: +90 342 3172189; Fax: +90 342 3604423; E-mail: hgcelikel@gantep.edu.tr (H.G. Sogukomerogullari); Tel: +90 246 211 0334 E-mail: senemakkoc@sdu.edu.tr (S. Akkoc)

Materials and Methods

All of the reactants and solvents were acquired from Aldrich or Merck (purity 95-99%) and used as received. The NMR spectrain DMSO-d_6 were measured on a Bruker High Performance Digital FT-NMR (400 MHz) spectrometer and TMS was used as an internal standard. A PG Instruments T80+UV/Vis spectrometer was used to plot UV-Vis spectra. The molar conductances were measured in DMF (10^{-3} M) at 25 °C using a Ohaus Starter 3100M model conductivity meter. The magnetic susceptibility of the complexes was measured on a Sherwood Scientific model Instrument. A Thermo Scientific Flash EA 2000 CHNS analyzer was used to perform the elemental analyses. A Perkin-Elmer Spectrum 100 FTIR spectrophotometer with an ATR sampling accessory was used to measure infrared spectra.

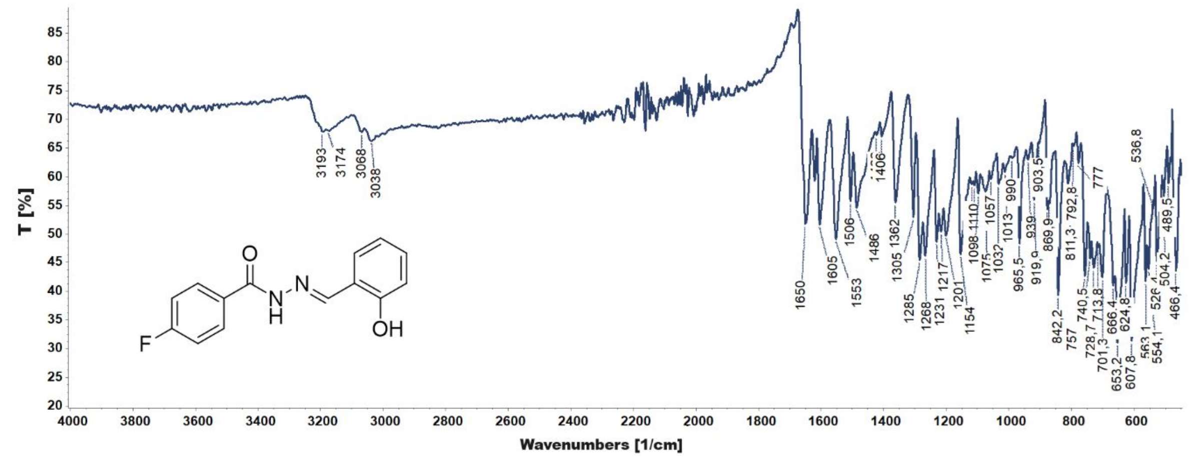

Figure S1. FT-IR spectrum of ligand.

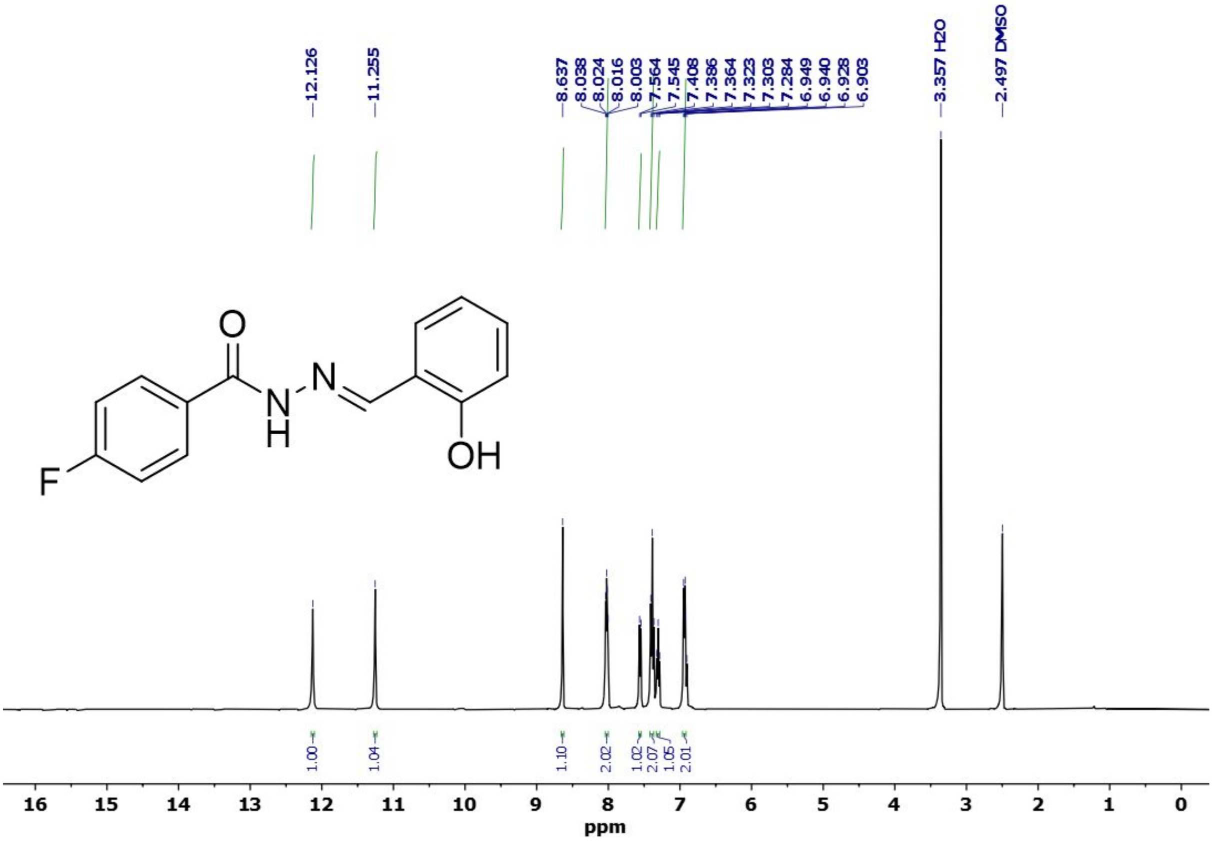

Figure S2. ^{1}H NMR spectrum of ligand.

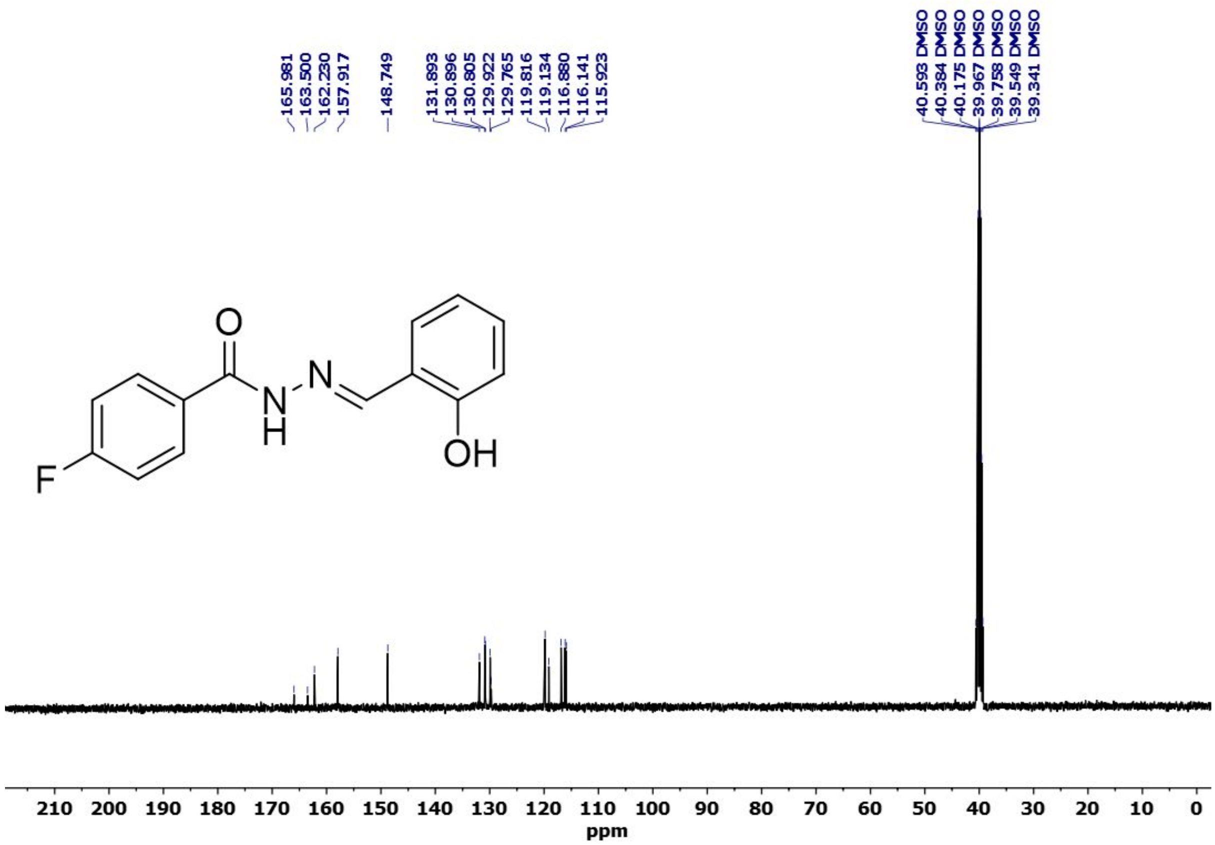

Figure S3. ^{13}C NMR spectrum of ligand.

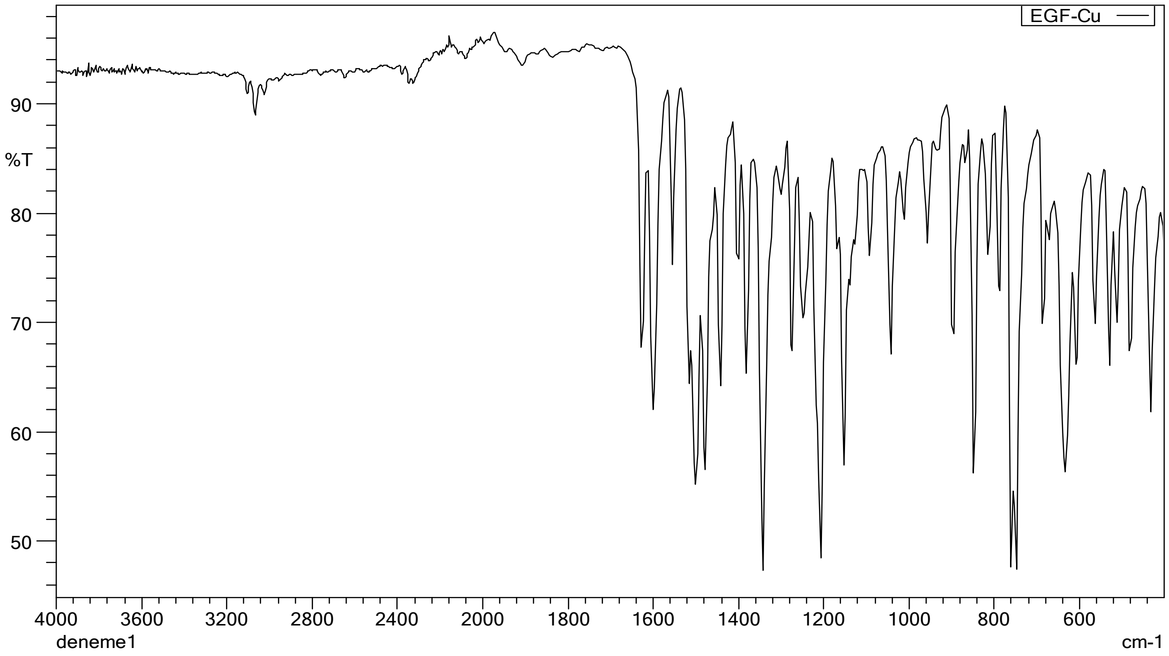

Figure S4. FT-IR spectrum of L-Cu.

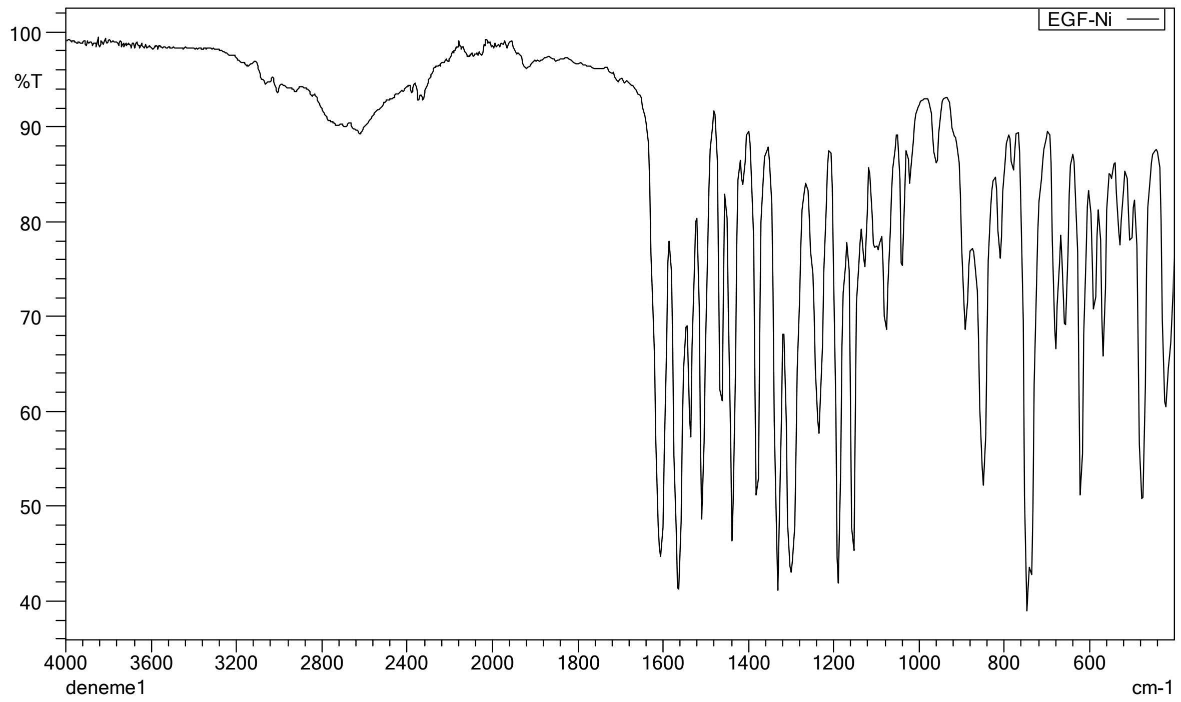

Figure S5. FT-IR spectrum of L-Ni.

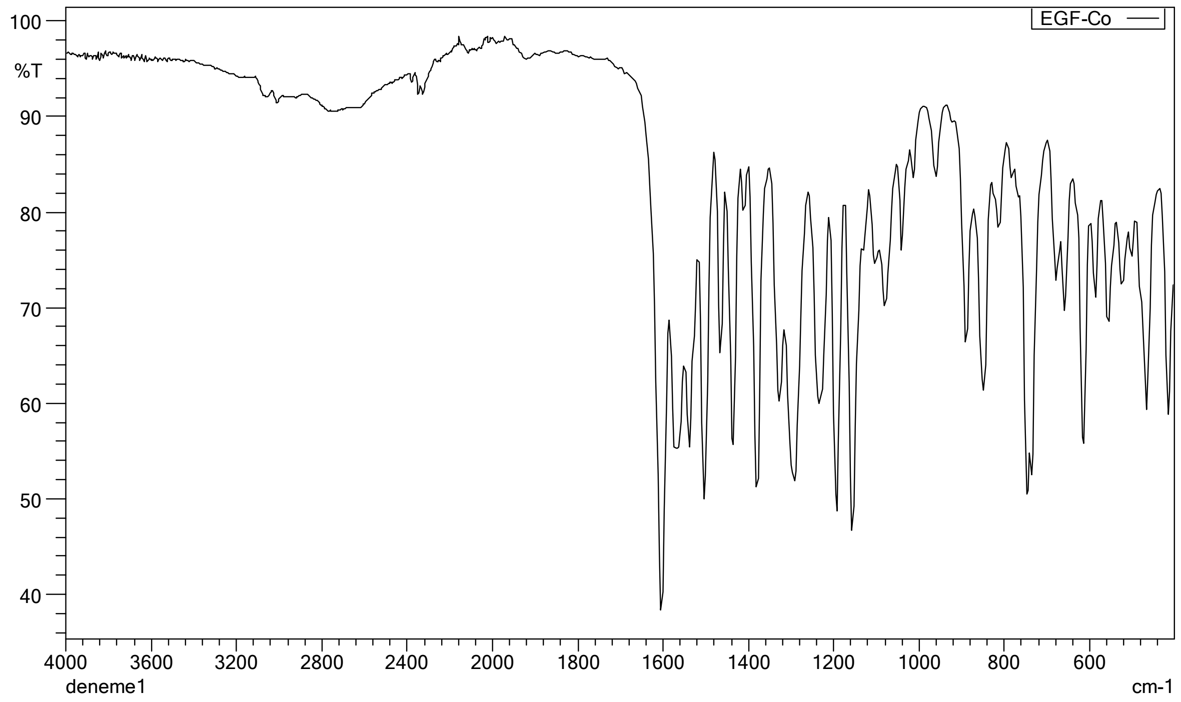

Figure S6. FT-IR spectrum of L-Co.

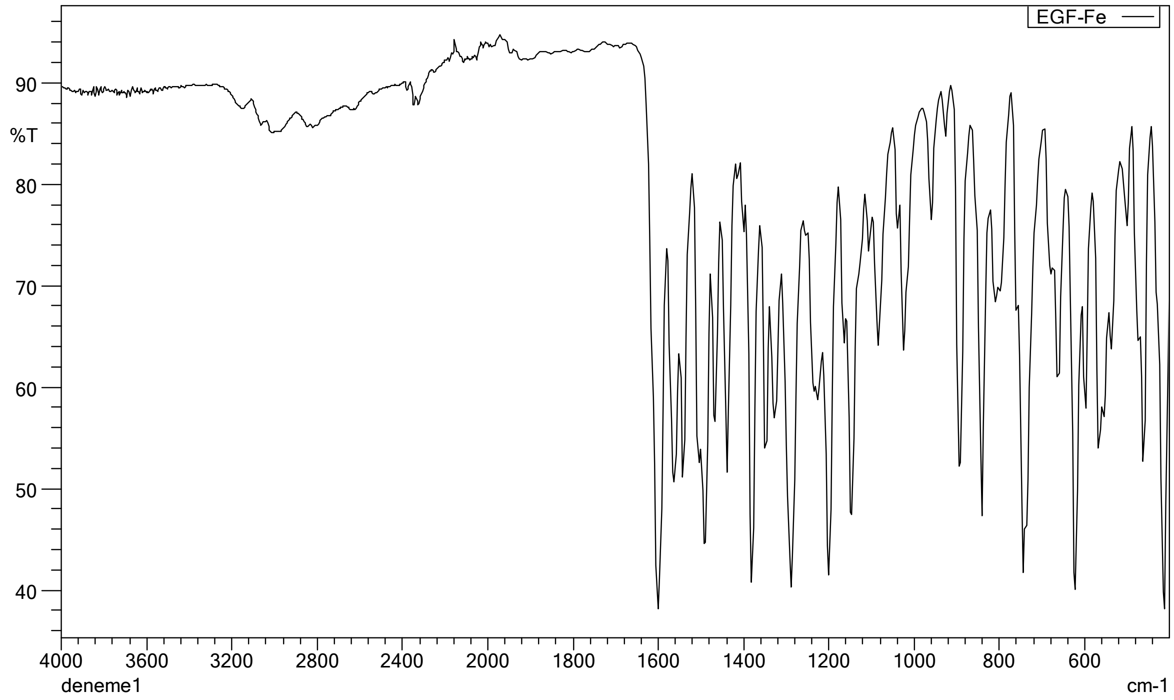

Figure S7. FT-IR spectrum of L-Fe.

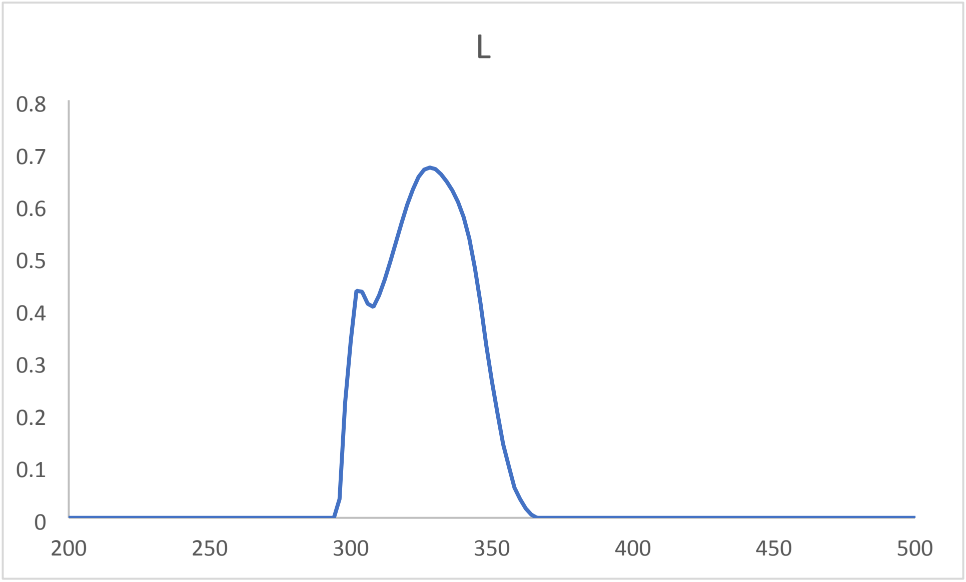

Figure S8. UV-Vis spectrum of ligand.

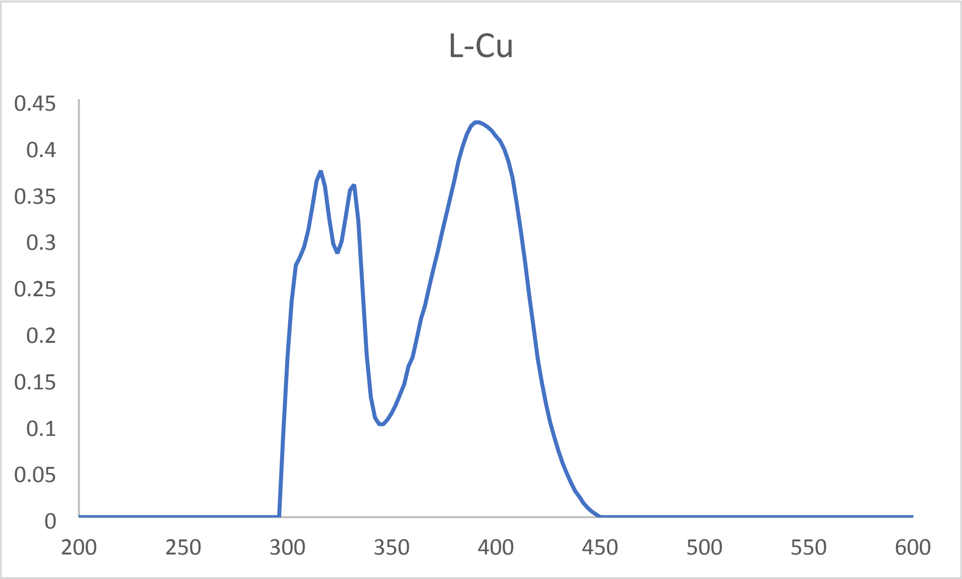

Figure S9. UV-Vis spectrum of L-Cu.

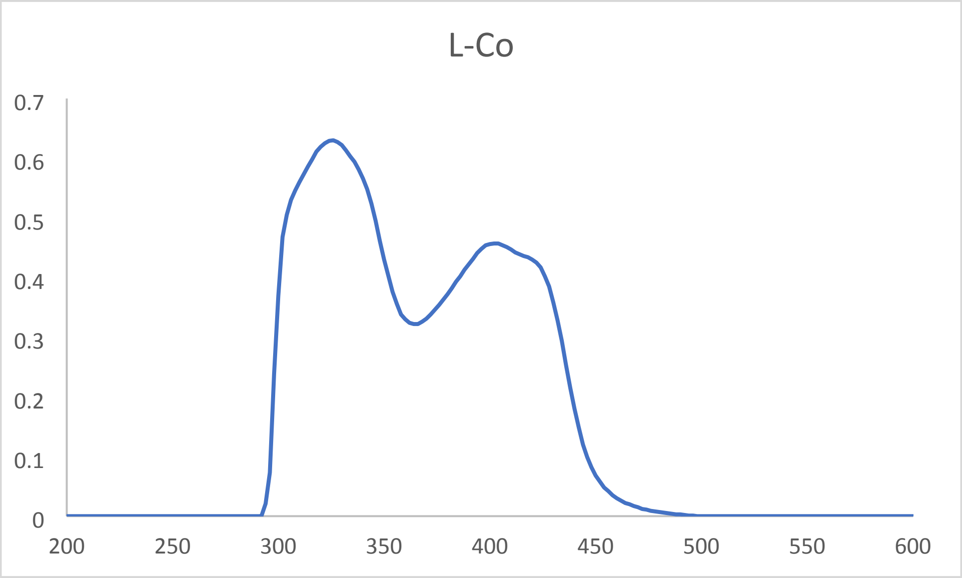

Figure S10. UV-Vis spectrum of L-Co.

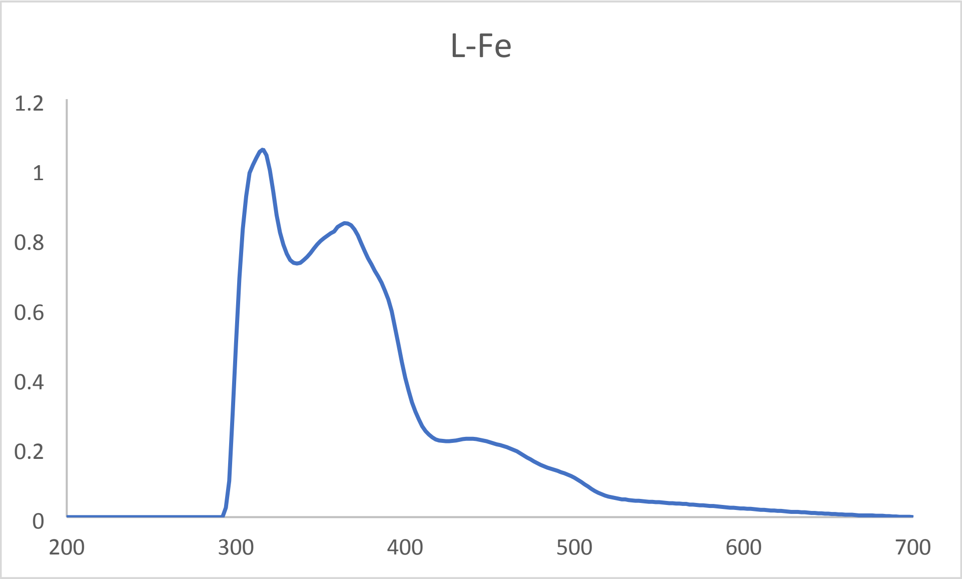

Figure S11. UV-Vis spectrum of L-Fe.

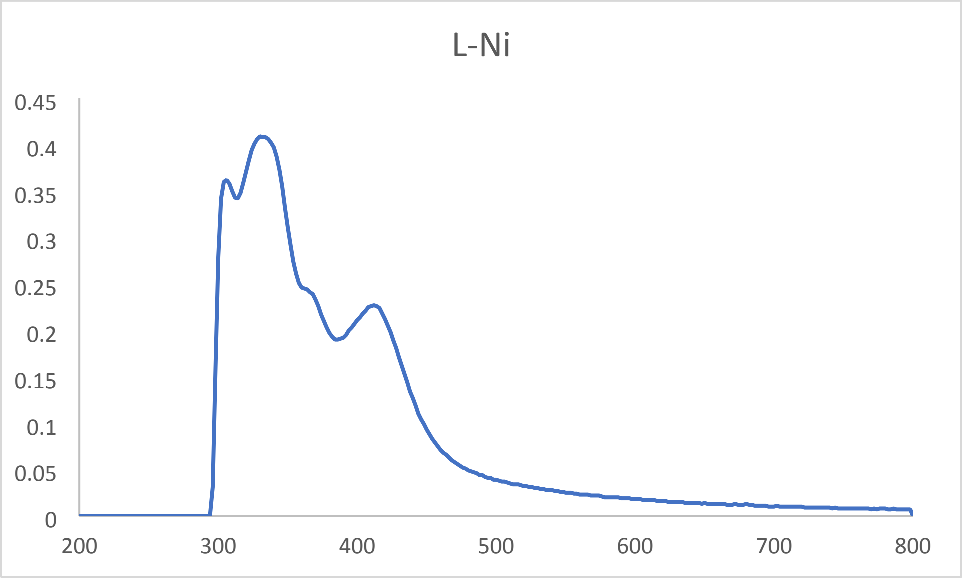

Figure S12. UV-Vis spectrum of L-Ni.
